# Discrimination and vigilance as psychosocial pathways from food insecurity to cognitive difficulty among U.S. adults: a moderated mediation analysis

**DOI:** 10.1186/s12889-026-26226-6

**Published:** 2026-01-13

**Authors:** Hyojin Lee, Hae Sagong

**Affiliations:** 1 Department of International Health, Johns Hopkins Bloomberg School of Public Health, Baltimore, MD USA; 2 College of Nursing, Auburn University, Auburn, AL USA

**Keywords:** Food insecurity, Discrimination, Vigilance, Cognition, Nativity, Health equity

## Abstract

**Background:**

Food insecurity is a growing public health concern associated with adverse cognitive outcomes and shaped by structural barriers. However, the psychosocial mechanisms linking food insecurity to cognitive difficulty remain underexplored. This study examined whether perceived discrimination and vigilance help explain this relationship and whether these pathways differ by U.S. nativity status.

**Methods:**

We used data from the 2023 National Health Interview Survey (NHIS) and analyzed 27,525 adults (23,000 U.S.-born and 4,525 foreign-born) aged 18 and older. Key variables included food insecurity, cognitive difficulty, perceived discrimination, and vigilance. We conducted moderated sequential mediation analyses, with discrimination and vigilance as mediators and nativity as a moderator. Models adjusted for age, sex, race/ethnicity, marital status, education, income, and self-rated health.

**Results:**

Food insecurity, discrimination, and vigilance were significantly associated with greater odds of cognitive difficulty. Among U.S.-born individuals, the sequential pathway from food insecurity through discrimination and vigilance was significant (β = 0.10, 95% CI: 0.08, 0.12), accounting for 52% of the total effect. Among foreign-born individuals, the pathway was also significant and fully mediated (β = 0.05, 95% CI: 0.03, 0.08), explaining 39% of the total effect. Nativity significantly moderated the full sequential pathway (β = -0.05, 95% CI: -0.07, -0.03), with stronger mediation observed among U.S.-born adults.

**Conclusions:**

Discrimination and vigilance help explain how food insecurity affects cognitive difficulty. Addressing both material hardship and psychosocial stress may help prevent cognitive decline across diverse populations.

**Supplementary Information:**

The online version contains supplementary material available at 10.1186/s12889-026-26226-6.

## Introduction

Food insecurity, defined as the limited or uncertain access to adequate and nutritious food, remains a persistent public health challenge in the United States [1]. In 2023, 13.5% of U.S. households (approximately 18.0 million) experienced food insecurity, with the highest prevalence occurring in nearly a decade [2]. In addition to nutritional consequences, food insecurity is increasingly recognized as a social stressor that undermines mental and cognitive health across the life course [3].

A substantial body of research links food insecurity to poorer cognitive function, including memory, attention, and executive function, all of which are critical for daily functioning and long-term well-being [4]. A U.S.-based longitudinal study reported that, compared with their food-secure counterparts, older adults who experienced food insecurity were 46% more likely to develop cognitive impairment and experienced significantly accelerated decline in cognitive function over six years [5]. The relationship between food insecurity and cognitive health is often explained through chronic stress, depression, or inadequate nutritional intake [6]. While recent studies have identified anxiety and depression as mediators [7, 8], these mental health factors explain only part of the relationship. Broader psychosocial pathways remain underexplored [3].

Although discrimination is commonly examined as a structural driver of food insecurity [9–11], experiencing food insecurity itself exacerbates perceived discrimination. Individuals experiencing food insecurity are disproportionately low-income and racially minoritized [12], groups that already face elevated discrimination on the basis of race and socioeconomic status [13]. Additionally, stigmatizing encounters when using the Supplemental Nutrition Assistance Program (SNAP) benefits or disclosing program participation further amplify exposure to discrimination [14]. These experiences create additional psychosocial stress that can harm cognitive health beyond nutritional deficits.

Discrimination rarely operates in isolation, as many individuals adopt vigilance (anticipatory monitoring and behavioral adjustment) as a coping response that develops following discriminatory experiences or in anticipation of their recurrence [15, 16]. According to cognitive load theory, sustained vigilance creates competing demands as individuals must simultaneously monitor their environment for threats while engaging in routine cognitive tasks [17, 18]. This divided attention leaves fewer cognitive resources available for memory consolidation and executive function, contributing to cognitive fatigue and difficulty over time [19].

Additionally, the attention-depletion hypothesis [20] posits that anticipatory stress redirects attention toward potential threats, reducing available resources for other cognitive processes and impairing inhibitory control [21], cognitive flexibility [22], and filtering efficiency [23]. These immediate attention deficits are compounded by prolonged activation of stress‒response pathways (e.g., the hypothalamic-pituitary-adrenal axis), which elevate glucocorticoids and can impair hippocampal and prefrontal regions critical for memory and executive control [24, 25]. Persistently high levels of these psychosocial stressors can increase allostatic load and diminish coping resources, which may be particularly detrimental among food-insecure individuals who face multiple, compounding stressors [26].

These processes may also differ by nativity, given the U.S.’s diverse immigrant population. While both U.S.-born and foreign-born individuals face discrimination and may engage in vigilance, their sources and experiences differ. U.S.-born individuals, particularly racial minorities, are more likely to encounter systemic racism rooted in U.S. history [27], whereas immigrants face discrimination related to language, documentation status, and acculturation [28].

Recent population-based evidence highlights that food insecurity varies across ethnic and nativity groups. For example, U.S.-born Hispanics and Blacks show higher odds of food insecurity than U.S.-born Whites, and recent immigrants (< 5 years) also exhibit elevated vulnerability [29]. A comparative study indicates that Mexican and West African immigrants experience lower food security than matched native households, whereas Chinese and Indian immigrants show higher food security, underscoring significant heterogeneity by origin [30]. In addition, Latino and Asian immigrants without legal permanent residency face disproportionately high food insecurity, highlighting how various factors may compound vulnerability [31].

Foreign-born individuals inherently face unique structural barriers to accessing food assistance, including eligibility restrictions, immigration-related fears, and limited awareness of available resources [14]. Cultural differences in food norms and immigration stress may also shape how food insecurity affects cognitive health [32]. Despite these challenges, the immigrant health paradox suggests that newcomers often arrive with better initial health outcomes due to selective migration and protective cultural factors, although these advantages may lessen with longer residence and acculturation [33]. Understanding these differences could reveal how structural inequities create distinct health vulnerabilities; however, these underlying mechanisms remain unclear. While discrimination and vigilance interact in complex ways, our model focuses on the pathway where discrimination precedes vigilance, consistent with prior studies [15, 16].

To address these gaps, this study uses a large, nationally representative sample of U.S. adults to examine whether general perceived discrimination and vigilance mediate the relationship between food insecurity and self-reported cognitive difficulty and whether these pathways differ by nativity. We hypothesize the following:

- Hypothesis H1. Food insecurity, discrimination, and vigilance will each be independently positively associated with cognitive difficulty.
- Hypothesis H2. Food insecurity will increase discrimination, which will increase vigilance, subsequently leading to cognitive difficulty.
- Hypothesis H3. The overall pattern linking food insecurity to cognitive difficulty through discrimination and vigilance will vary by nativity status, given mixed evidence in prior literature.

## Methods

### Data sources and study population

We used data from the 2023 National Health Interview Survey (NHIS), a nationally representative cross-sectional household survey. The NHIS employs a complex, multistage probability sampling design across all 50 states and the District of Columbia. From each selected household, at least one member aged 18 years or older was randomly chosen to provide detailed socioeconomic and health information. The survey includes the civilian noninstitutionalized population, excluding active-duty military personnel, institutionalized individuals, and those without fixed addresses. The 2023 dataset included 29,522 adults, with a final response rate of 47.0%. To address potential nonresponse bias, all analyses incorporated NHIS final annual weights, which included base sampling weights, nonresponse adjustments using classification tree methods, and post-stratification calibration to U.S. Census population controls. After excluding cases with missing data on the study variables (*n* = 1,997; 6.8%), our final analytic sample comprised 27,525 adults.

### Measures

#### Cognitive difficulty

The primary outcome was self-reported cognitive difficulty, assessed via the cognition domain from the Washington Group Short Set on Functioning (WG-SS) [34]: “Do you have difficulty remembering or concentrating?” Response options were four ordered categories: “no difficulty” (79.6%), “some difficulty” (17.7%), “a lot of difficulty” (2.6%), and “cannot do at all” (0.1%). For analysis, we dichotomized the item into “no difficulty” versus “any difficulty” by combining the latter three categories, due to sparse counts in the severe categories, consistent with prior research [7].

#### Food insecurity

Food insecurity was assessed via the 10-item USDA Food Security Survey Module, which examines household food access and affordability over the past 30 days [35]. On the basis of affirmative responses, food security was classified into four levels: high (0 items, 84.6%), marginal (1–2 items, 6.4%), low (3–5 items, 5.1%), and very low (≥ 6 items, 3.9%). Following USDA categorization, the responses were dichotomized into “food secure” (high/marginal) and “food insecure” (low/very low).

#### Perceived discrimination

Perceived discrimination was measured via five items from the short version of the Everyday Discrimination Scale (EDS) [36]. The participants were asked how often they encountered five types of unfair treatment over the past 12 months, including being treated with less courtesy or respect, receiving poorer service in public settings, being perceived as not smart, having others act afraid of them, or feeling threatened or harassed. The response options ranged from “never” (0) to “at least once a week” (4); the total scores ranged from 0 to 20, with higher scores indicating more frequent perceived discrimination. The scale demonstrated acceptable reliability (Cronbach’s α = 0.73).

#### Vigilance

Vigilance was assessed via the 4-item Heightened Vigilance Scale [15]. The participants were asked how frequently they engaged in anticipatory behaviors, such as preparing for insults, avoiding certain places, or modifying their appearance or speech to prevent mistreatment. The response scale was identical to that used in the EDS (0–4); total scores ranged from 0 to 16, with higher scores indicating greater vigilance. Internal consistency was acceptable (Cronbach’s α = 0.71).

#### Nativity

Nativity was derived from the question, “Were you born in the United States or a U.S. territory?” Responses of “yes” were categorized as U.S.-born, and “no” were categorized as foreign-born.

#### Covariates

The covariates included age, sex, race/ethnicity, marital status, education, income, and self-rated health [7]. The categories were as follows: age (18–39, 40–64, and ≥ 65 years); sex (male and female); race/ethnicity (non-Hispanic [NH] White, Hispanic, NH Black, NH Asian, and other, including American Indian/Alaska Native [AIAN] and multiracial individuals); marital status (partnered and unpartnered); education (< high school, high school, some college/associate and ≥ bachelor’s); income (family income‒poverty ratio); and general health (excellent, good, and fair/poor).

### Data analysis

Analyses were conducted using Stata/BE 18.0 software (Stata Corp, College Station, TX) and SPSS 29.0 PROCESS macro 4.5 (IBM Corporation, Chicago, IL). Descriptive statistics included unweighted counts and survey-weighted means or percentages. All models accounted for the complex sampling design of the NHIS. To examine this association, we conducted hierarchical logistic regression analyses with cognitive difficulty as the outcome: Model 1 included food insecurity (primary exposure) and covariates; Model 2 further included discrimination (first mediator); and Model 3 further included vigilance (second mediator). Nativity served as the moderator, with analyses stratified by U.S.-born versus foreign-born status. Adjusted odds ratios (aORs) with 95% confidence intervals (CIs) are reported. Indirect and sequential effects were tested via moderated serial mediation (PROCESS Models 6 and 83), stratified by nativity. Bias-corrected 95% CIs were estimated via 5,000 bootstrap resamples.

Given the binary outcome (cognitive difficulty), paths to this outcome were estimated with log-odds coefficients, whereas paths between continuous mediators used linear regression coefficients. Although discrimination and vigilance were moderately correlated (*r* = .60), this was expected given our conceptual model in which discrimination triggers vigilance; variance inflation factors (VIFs) below 2.5 confirmed that there were no concerns of multicollinearity. Missing data were minimal (< 7%) and examination of missingness patterns revealed no systematic associations. We used listwise deletion, which yields unbiased estimates when data are missing completely at random and missingness is minimal [37]. Statistical significance was set at *p* < .05.

## Results

Table 1 presents participant characteristics by nativity. Nativity group differences were tested using survey-weighted Pearson χ² tests for categorical variables and t-tests for continuous variables. All comparisons were statistically significant (*p* < .05). The sample consisted of 23,000 (81.3%) U.S.-born adults and 4,525 (18.7%) foreign-born adults. U.S.-born participants were more likely to be younger and NH White (73.1%), whereas foreign-born participants were more likely to be middle-aged and Hispanic (47.1%) or NH Asian (25.1%). Compared with U.S.-born adults, foreign-born adults had a lower income-to-poverty ratio (3.6 vs. 4.3) and slightly higher food insecurity (10.6% vs. 8.5%). They also reported lower cognitive difficulty (14.5% vs. 21.8%) and lower mean levels of perceived discrimination (1.8 vs. 2.6) and vigilance (2.9 vs. 3.5).

**Table 1:** Participant characteristics by nativity status (*N* = 27,525)

	Total	U.S.-born *n* = 23,000 (81.3%)	Foreign-born *n* = 4,525 (18.7%)	χ² /t (*p*)
	*n* (%) or Mean (SD)			
Age				269.18 (< 0.001)
18–39	7,914 (37.8%)	6,557 (39.1%)	1,357 (32.3%)	
40–64	10,500 (39.4%)	8,391 (37.1%)	2,109 (49.5%)	
≥ 65	9,111 (22.8%)	8,052 (23.8%)	1,059 (18.3%)	
Sex				6.10 (0.042)
Male	12,563 (48.9%)	10,522 (49.2%)	2,041 (47.3%)	
Female	14,962 (51.1%)	12,478 (50.8%)	2,484 (52.7%)	
Race/ethnicity				9078.79 (< 0.001)
NH White	18,407 (62.7%)	17,467 (73.1%)	940 (17.5%)	
Hispanic	4,064 (17.3%)	2,075 (10.4%)	1,989 (47.1%)	
NH Black	2,863 (11.3%)	2,493 (11.8%)	370 (9.0%)	
NH Asian	1,521 (6.2%)	357 (1.8%)	1,164 (25.1%)	
Other	620 (2.5%)	608 (2.8%)	62 (1.3%)	
Marital status				244.12 (< 0.001)
Partnered	14,460(60.3%)	11,624 (58.1%)	2,836 (69.9%)	
Unpartnered	13,065 (39.7%)	11,376 (41.9%)	1,689 (30.1%)	
Education				1010.21 (< 0.001)
< High school	2,935 (12.5%)	2,006 (9.7%)	929 (24.6%)	
High school graduate	6,321 (24.5%)	5,422 (25.2%)	899 (21.6%)	
Some college/associate	7,676 (29.6%)	6,871 (32.0%)	805 (19.0%)	
≥ Bachelor’s degree	10,593 (33.4%)	8,701 (33.2%)	1,892 (34.7%)	
Income poverty ratio (0–11)	4.1 (2.9)	4.3 (3.0)	3.6 (3.0)	-8.56 (< 0.001)
General health				28.99 (< 0.001)
Excellent	14,988 (56.1%)	12,563 (56.8%)	2,425 (53.2%)	
Good	8,124 (29.1%)	6,719 (28.4%)	1,405 (32.1%)	
Fair/poor	4,413 (14.8%)	3,718 (14.8%)	695 (14.7%)	
Food insecurity				21.76 (< 0.001)
Food secure	25,160 (91.1%)	21,091 (91.5%)	4,069 (89.4%)	
Food insecure	2,365 (8.9%)	1,909 (8.5%)	456 (10.6%)	
Cognitive difficulty				74.12 (< 0.001)
No difficulty	21,602 (79.6%)	17,755 (78.2%)	3,847 (85.5%)	
Any difficulty	5,923 (20.4%)	5,245 (21.8%)	678 (14.5%)	
Discrimination (0–20)	2.5 (3.3)	2.6 (3.4)	1.8 (2.9)	-12.02 (< 0.001)
Vigilance (0–16)	3.4 (4.0)	3.5 (4.0)	2.9 (3.8)	-8.12 (< 0.001)

Note. *n* is unweighted; % and mean (SD) are weighted

*NH* Non-Hispanic

Despite the statistical differences, effect sizes were generally small (Cohen’s d and Cramér’s V < 0.20), with the exception of race/ethnicity (V = 0.57). Small-to-moderate effects were observed for discrimination (d = 0.19) and education (V = 0.16), indicating that the practical magnitude of most differences was limited.

To examine whether food insecurity was associated with heightened psychosocial stressors, we compared the mean (M) levels of discrimination and vigilance by food security status, stratified by nativity (Table 2). In both the U.S.-born and foreign-born groups, individuals with food insecurity reported significantly higher levels of discrimination and vigilance (all *p* < .001). Among all the subgroups, U.S.-born adults with food insecurity reported the highest levels of discrimination (M = 4.9) and vigilance (M = 6.1).

**Table 2:** Psychosocial stress indicators by food security status and nativity (*N* = 27,525)

Variables	U.S.-born (*n* = 23000)			Foreign-born (*n* = 4525)		
	Secure (*n* = 21,091)	Insecure (*n* = 1,909)	*p*	Secure (*n* = 4,069)	Insecure (*n* = 456)	*p*
Discrimination (0–20)	2.4 (3.2)	4.9 (4.4)	< 0.001	1.7 (2.7)	2.7 (4.1)	< 0.001
Vigilance (0–16)	3.3 (3.8)	6.1 (4.9)	< 0.001	2.7 (3.7)	3.8 (4.5)	< 0.001

Note. *n* unweighted; values are weighted means (SD)

We also explored variation in stressor levels across racial/ethnic groups within nativity status (see Supplementary Table 1). Among U.S.-born adults, NH Black individuals reported the highest overall mean discrimination (M = 4.0) and vigilance (M = 5.2), which increased substantially among food-insecure NH Black individuals (discrimination M = 5.7; vigilance M = 7.0). In contrast, NH White individuals, despite comprising 73% of the U.S.-born sample, reported relatively lower mean stressor levels. No significant differences by food security status were observed for NH Asian adults. Among foreign-born adults, those categorized as “Other” reported the highest stressor levels when food insecurity was present (discrimination M = 5.8; vigilance M = 6.7), followed by NH Black individuals (discrimination M = 4.8; vigilance M = 5.4). Differences by food security status were not significant for foreign-born NH Asian or NH White adults.

To test H1, we conducted hierarchical logistic regression models to examine the independent effects of food insecurity, discrimination, and vigilance on cognitive difficulty, adjusting for covariates (Table 3). Analyses used the pooled sample to identify overall associations before mediation, with nativity included as a covariate. In Model 1, food insecurity was associated with 83% higher odds of cognitive difficulty (aOR = 1.83, 95% CI = 1.62, 2.06). This association was attenuated by 42% after adding discrimination in Model 2 (aOR = 1.48, 95% CI = 1.30, 1.69) and by 53% after further adding vigilance in Model 3, though it remained statistically significant (aOR = 1.39, 95% CI = 1.22, 1.59). Both discrimination (aOR = 1.07, 95% CI = 1.06, 1.09) and vigilance (aOR = 1.08, 95% CI = 1.07, 1.10) were independently associated with cognitive difficulty, supporting H1.

**Table 3:** Hierarchical logistic regression models predicting cognitive difficulty (pooled sample, *N* = 27,525)

Variables	Model 1	Model 2	Model 3
	aOR (95% CI)	aOR (95% CI)	aOR (95% CI)
Food insecurity	1.83^***^ (1.62, 2.06)	1.48^***^ (1.30, 1.69)	1.39^***^ (1.22, 1.59)
Discrimination	—	1.13^***^ (1.12, 1.14)	1.07^***^ (1.06, 1.09)
Vigilance	—	—	1.08^***^ (1.07, 1.10)
Age			
18–39	Ref.		
40–64	0.70^***^ (0.64, 0.77)	0.75^***^ (0.68, 0.82)	0.78^***^ (0.71, 0.86)
≥ 65	1.26^***^ (1.15, 1.39)	1.61^***^ (1.46, 1.78)	1.76^***^ (1.59, 1.95)
Sex			
Male	Ref.		
Female	1.24^***^ (1.15, 1.33)	1.24^***^ (1.15, 1.34)	1.23^***^ (1.14, 1.33)
Race/ethnicity			
NH White	Ref.		
Hispanic	0.72^***^ (0.62, 0.82)	0.72^***^ (0.63, 0.83)	0.71^***^ (0.61, 0.81)
NH Black	0.66^***^ (0.58, 0.75)	0.55^***^ (0.48, 0.63)	0.52^***^ (0.45, 0.59)
NH Asian	0.66^***^ (0.54, 0.82)	0.64^***^ (0.52, 0.80)	0.63^***^ (0.50, 0.79)
Other	0.89 (0.70, 1.13)	0.79 (0.62, 1.02)	0.77^***^ (0.61, 0.99)
Nativity			
US-born	Ref.		
Foreign-born	0.70^***^ (0.61, 0.81)	0.77^***^ (0.67, 0.89)	0.78^***^(0.68, 0.89)
Marital status			
Unpartnered	Ref.		
Partnered	0.78^***^ (0.72, 0.85)	0.80^***^ (0.74, 0.87)	0.81^***^ (0.74, 0.87)
Education	0.93^**^ (0.89, 0.97)	0.90^***^ (0.87, 0.94)	0.89^***^ (0.85, 0.92)
Poverty	0.96^***^ (0.94, 0.97)	0.95^***^ (0.94, 0.97)	0.95^***^ (0.94, 0.97)
General health			
Excellent	Ref.		
Good	2.13^***^ (1.95, 2.32)	2.01^***^ (1.84, 2.19)	1.98^***^ (1.82, 2.16)
Fair/poor	4.70^***^ (4.24, 5.20)	4.29^***^ (3.87, 4.76)	4.21^***^ (3.80, 4.68)
Overall F (df)	148.67^***^ (14, 597)	162.41^***^ (15, 596)	167.70^***^ (16, 595)

Note. *aOR* Adjusted odds ratio, *CI* Confidence interval, *NH* Non-Hispanic

The reference group for food insecurity is food security. Education is treated as an ordinal variable. ^***^p < .001 ^**^p < .01

**Fig. 1:**
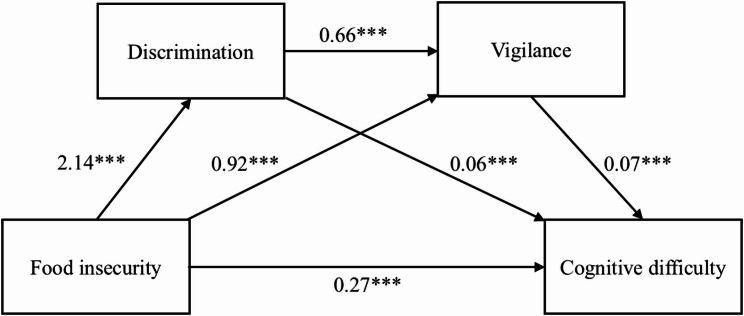
Sequential mediation model for U.S.-born participants. Note. Path coefficients represent unstandardized regression coefficients; paths to cognitive difficulty are log-odds values. ***p < .001

**Fig. 2:**
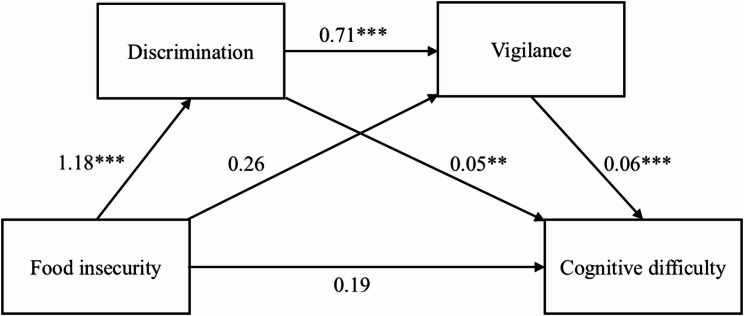
Sequential mediation model for foreign-born participants. Note. Path coefficients represent unstandardized regression coefficients; paths to cognitive difficulty are log-odds values. Nonsignificant paths are shown without asterisks. ***p < .001; **p < .01

To test H2, we examined the sequential mediation linking food insecurity to cognitive difficulty through discrimination and vigilance within each nativity group (see Figs. 1 and 2; Table 4). Path coefficients shown in Figs. 1 and 2 represent unstandardized log-odds values from the mediation models, which differ from the aORs reported in Table 3.

**Table 4:** Sequential mediation analysis with moderation by nativity status

Pathway	U.S.-born (*n* = 23,000)					Foreign-born (*n* = 4,525)			
**Direct effect**	β	SE		95% CI		β	SE		95% CI
FI → CD	**0.27**	0.06		(0.16, 0.38)		0.19	0.13		(-0.07, 0.45)
**Indirect effects**	Effect	Boot SE		Boot 95% CI		Effect	Boot SE		Boot 95% CI
FI → Discrimination → CD	**0.13**	0.01		(0.10, 0.16)		**0.05**	0.02		(0.01, 0.10)
FI → Vigilance → CD	**0.07**	0.01		(0.05, 0.08)		0.02	0.01		(-0.01, 0.04)
FI → Discrimination → Vigilance → CD	**0.10**	0.01		(0.08, 0.12)		**0.05**	0.01		(0.03, 0.08)
Total indirect effect	**0.30**	0.02		(0.26, 0.34)		**0.12**	0.03		(0.07, 0.17)
**Percent mediated (%)**	52.4					39.0			
**Moderated mediation index**	Effect					Boot SE			Boot 95% CI
FI → Discrimination → CD	**-0.07**					0.02			(-0.10, -0.04)
FI → Discrimination → Vigilance → CD	**-0.05**					0.01			(-0.07, -0.03)

Note. *FI*  Food insecurity, *CD*  Cognitive difficulty, β represents log odds

Bold values are significant at *p* < .05

Among U.S.-born adults, food insecurity was significantly associated with higher discrimination (β = 2.14, *p* < .001), greater vigilance (β = 0.92, *p* < .001), and cognitive difficulty (β = 0.27, *p* < .001). Discrimination also predicted greater vigilance (β = 0.66, *p* < .001). Both discrimination (β = 0.06, *p* < .001) and vigilance (β = 0.07, *p* < .001) were in turn associated with greater cognitive difficulty. The sequential indirect effect (food insecurity → discrimination → vigilance → cognitive difficulty) was β = 0.10 (95% CI = 0.08, 0.12), contributing to a total indirect effect of β = 0.30 (95% CI = 0.26, 0.34). The direct path remained significant, indicating partial mediation.

Among foreign-born adults, food insecurity predicted higher discrimination (β = 1.18, *p* < .001). In turn, discrimination predicted greater vigilance (β = 0.71, *p* < .001) and was associated with cognitive difficulty (β = 0.05, *p* < .01), while vigilance was also associated with cognitive difficulty (β = 0.06, *p* < .001). However, the direct path from food insecurity to vigilance was not significant after accounting for discrimination. The sequential indirect effect was β = 0.05 (95% CI = 0.03, 0.08), contributing to a total indirect effect of β = 0.12 (95% CI = 0.07, 0.17). The nonsignificant direct effect (β = 0.19, 95% CI = -0.07, 0.45) suggests full mediation. Taken together, these findings support H2.

To test H3, we compared mediation strength across nativity groups. U.S.-born adults showed stronger mediation (52.4% mediated) compared to foreign-born adults (39.0% mediated). The significant moderated mediation index (β = -0.05, 95% CI = -0.07, -0.03) confirmed that these psychosocial pathways varied by nativity status, with stronger overall mediation observed among U.S.-born adults. These results support H3.

## Discussion

This study provides evidence that discrimination and vigilance mediate the relationship between food insecurity and cognitive difficulty among U.S. adults, with substantial variation by nativity status. Using a nationally representative sample and moderated mediation analysis, we found that food insecurity was associated with higher perceived discrimination and vigilance, which were in turn related to cognitive difficulty.

In the pooled sample, food insecurity was associated with higher odds of cognitive difficulty, consistent with prior research [4, 38]. This association appeared more pronounced among U.S.-born adults despite their lower food insecurity prevalence. However, a study that disaggregated immigrants by citizenship and duration reported stronger associations for certain immigrant subgroups [39], indicating considerable heterogeneity within foreign-born populations. For example, among Asian immigrants in California, food insecurity ranged from 2.3% to 16.4% depending on racial/ethnic community and acculturation level [40]. Consistent with this variability, our subgroup analyses showed heterogeneous patterns across racial/ethnic groups. Among foreign-born adults, NH Asian and NH White groups exhibited no significant differences in discrimination or vigilance by food security status, suggesting that the food insecurity–psychosocial stress pathway may not operate uniformly across immigrant subgroups. Structural barriers such as documentation status and cultural stigma further shape access to food assistance [40, 41]. Such variation underscores the complexity of immigrant experiences with food insecurity, highlighting the need for future research to disaggregate nativity (e.g., by citizenship, duration in the U.S., and origin) and examine how intersecting identities influence the food insecurity–cognition relationship over time.

Racial disparities further contextualize the stronger associations observed among U.S.-born adults. NH Black individuals reported the highest levels of perceived discrimination and vigilance despite their smaller share of the U.S.-born sample, whereas NH White individuals, who comprised the majority group, reported comparatively lower stressor levels. This pattern indicates that the racialized stress among minoritized groups likely drives the overall associations. This disproportionate burden aligns with prior research showing that Black Americans experience chronic exposure to systemic racism and marginalization, which contributes to sustained psychosocial stress and cognitive strain [42, 43]. Discrimination has also been linked to poorer episodic memory performance among Black adults [44–46]. Additionally, the younger age profile of the U.S.-born sample may influence these findings. Younger generations tend to be more attuned and sensitive to social inequities and more likely to recognize and voice experiences of discrimination [47, 48]. This, in turn, may underlie the elevated stress levels and stronger patterns observed in this group.

In both nativity groups, discrimination and vigilance were significantly associated with cognitive difficulty, underscoring their importance as psychosocial correlates of cognitive health. Among U.S.-born adults, experiences of discrimination may directly impair cognitive function through emotional distress, rumination, and mental preoccupation with unfair treatment [49]. At the same time, chronic vigilance, whether stemming from discrimination or other perceived threats, can deplete attentional and emotional resources needed for concentration and memory. While discrimination and vigilance are interconnected, as individuals who experience more frequent discrimination also report heightened vigilance, both independently contributed to cognitive difficulty. However, discrimination and vigilance only partially mediated the food insecurity–cognition association; a substantial direct effect remained. This pattern suggests that additional pathways—such as nutritional deficiencies, financial strain, housing instability, or comorbid health conditions—may also link food insecurity to cognitive vulnerability in this population [3, 6].

In contrast, among foreign-born adults, discrimination and vigilance fully mediated the food insecurity–cognition association, underscoring their centrality as pathways to cognitive difficulty. The discrimination-to-vigilance pathway appeared particularly critical in this group, as food insecurity triggered vigilance primarily through experiences of discrimination rather than directly. This sequential pattern may reflect migration-specific contexts where discrimination serves as the primary gateway activating vigilant coping responses. Navigating unfamiliar sociocultural environments, documentation concerns, and language barriers may intensify how discriminatory experiences translate into heightened vigilance [50]. Prior trauma or instability in premigration contexts may further sensitize immigrants to subsequent stress exposures, heightening threat perception and amplifying vigilance when exclusion or bias is perceived [51].

Although the association was fully mediated in foreign-born adults, the overall magnitude of these indirect pathways was smaller than in U.S.-born adults. This pattern likely reflects lower cognitive difficulty prevalence in foreign-born adults. While migration-related stressors can amplify discrimination and vigilance impacts, protective cultural factors also appear to buffer cognitive effects, consistent with the immigrant health paradox framework [33]. For example, coethnic networks, pre-migration resources, and cultural coping strategies help mitigate vulnerability to food insecurity. Additionally, some immigrants do not readily recognize or label certain experiences as discrimination [52], instead attributing them to language barriers or cross-cultural misunderstandings. Different cultural expectations regarding food quantity and quality may also influence how food insecurity is perceived as threatening, thereby dampening its downstream effects. However, such cultural protective effects often erode with longer U.S. residence, as cumulative exposure to systemic inequities leads to health convergence with U.S.-born individuals from similar racial/ethnic backgrounds [53].

Given these nativity-specific patterns, tailored approaches may be warranted. For U.S.-born adults, particularly racially minoritized groups, interventions should address both food insecurity and chronic psychosocial stressors, as these only partially explained cognitive impacts. For foreign-born adults, strategies that reduce discriminatory experiences in food assistance settings and provide culturally sensitive support may be particularly effective. However, because many households include both U.S.-born and foreign-born members, programs should adopt flexible, integrated approaches rather than siloed programs targeting only one group. Additionally, given the protective role of cultural factors among foreign-born adults, interventions should aim to preserve and strengthen existing community resources that buffer stress impacts.

These recommendations align with emerging evidence for culturally tailored interventions [54–56]. However, most existing programs address food insecurity and cognitive health separately, without considering these mediating psychosocial pathways. To translate these findings into practice, coordinated action is needed across sectors. Researchers should test whether interventions that simultaneously address food insecurity and reduce discriminatory experiences can more effectively mitigate cognitive risk by disrupting these pathways. Policymakers should consider anti-discrimination measures in food assistance programs, such as training for service providers and oversight mechanisms. Healthcare systems should routinely screen for food insecurity as a cognitive health risk factor, with particular attention to the distinct pathways operating in immigrant versus U.S.-born populations.

This study has several limitations. First, the cross-sectional design precludes causal inference. The mediation models assume a temporal sequence that cannot be established with cross-sectional data, and reverse causation is plausible. Secondary data analysis also limited our ability to include key contextual factors such as neighborhood context and healthcare access. Second, reliance on English/Spanish surveys likely excluded immigrants with limited proficiency in these languages, potentially underrepresenting vulnerable populations. Self-reported cognitive difficulty lacks clinical precision and may reflect cultural differences in symptom perception. Third, our quantitative approach could not capture lived experiences navigating food insecurity, perceived discrimination, and vigilance responses. Fourth, we treated foreign-born adults as a single group, which masks heterogeneity in country of origin, migration history, and acculturation that may differentially shape experiences of food insecurity and psychosocial stress. Future research should employ longitudinal designs, validated cognitive assessments, multilingual data collection, stratified analyses by migration characteristics, and mixed-methods approaches to better understand immigrant experiences and cultural nuances. Intersectional analyses examining how nativity, race/ethnicity, and other social identities intersect are also needed to inform equity-focused interventions.

## Conclusions

This study demonstrates that discrimination and vigilance mediate the associations between food insecurity and cognitive difficulty through distinct nativity-specific pathways. These findings reveal that food insecurity affects cognitive health not only through material deprivation but through psychosocial mechanisms involving discriminatory experiences and vigilance. Addressing cognitive health disparities in food-insecure populations requires integrated, tailored interventions that target both food access and the psychosocial stressors activated by food insecurity across diverse populations.

## Supplementary Information

Supplementary Material 1.

## Data Availability

The dataset used and analyzed during the current study is publicly available through the National Health Interview Survey (NHIS) website: https://www.cdc.gov/nchs/nhis/documentation/2023-nhis.html.
