# Supplementary material for: Discrimination and vigilance as psychosocial pathways from food insecurity to cognitive difficulty among U.S. adults: a moderated mediation analysis
Source: BMC Public Health. 2026 Jan 13;26:537. doi: 10.1186/s12889-026-26226-6 (PMC12888570; doi:10.1186/s12889-026-26226-6)
Supplement: Supplementary file 1 — Supplementary Material 1. [file 12889_2026_26226_MOESM1_ESM.docx]

**Supplementary Table 1** Psychosocial stress indicators by food security, nativity, and race/ethnicity (*N* = 27,525)

| Variable | Race/  ethnicity | U.S.-born (*n* = 23,000) | | | | Foreign-born (*n* = 4,525) | | | |
| --- | --- | --- | --- | --- | --- | --- | --- | --- | --- |
|  |  | Total | Secure  (*n* = 21,091) | Insecure  (*n* = 1,909) | *p* | Total | Secure  (*n* = 4,069) | Insecure  (*n* = 456) | *p* |
| Discrimination | NH White | 2.3 (3.2) | 2.2 (3.0) | 4.7 (4.4) | <.001 | 1.9 (2.9) | 1.9 (2.9) | 2.3 (3.3) | 0.163 |
|  | Hispanic | 2.7 (3.3) | 2.5 (3.1) | 4.1 (4.2) | <.001 | 1.6 (2.5) | 1.5 (2.3) | 2.3 (3.5) | <.001 |
|  | NH Black | 4.0 (4.1) | 3.7 (3.9) | 5.7 (5.0) | <.001 | 3.0 (3.6) | 2.6 (3.3) | 4.8 (4.2) | 0.001 |
|  | NH Asian | 3.0 (3.3) | 3.0 (3.3) | 3.5 (2.5) | 0.440 | 1.8 (2.5) | 1.8 (2.4) | 2.1 (3.2) | 0.122 |
|  | Other | 3.6 (3.9) | 3.2 (3.6) | 5.7 (4.7) | <.001 | 3.0 (3.5) | 2.6 (2.9) | 5.8 (5.4) | 0.013 |
| Vigilance | NH White | 3.1 (3.8) | 2.9 (3.6) | 5.8 (4.9) | <.001 | 2.7 (3.7) | 2.7 (3.7) | 2.0 (3.3) | 0.654 |
|  | Hispanic | 3.7 (4.0) | 3.4 (3.8) | 5.7 (5.0) | <.001 | 2.7 (3.3) | 2.5 (3.2) | 3.7 (4.0) | <.001 |
|  | NH Black | 5.2 (4.7) | 4.9 (4.6) | 7.0 (5.2) | <.001 | 4.0 (4.2) | 3.7 (4.0) | 5.4 (4.7) | 0.013 |
|  | NH Asian | 4.1 (3.9) | 4.1 (4.0) | 4.7 (3.2) | 0.711 | 2.8 (3.4) | 2.8 (3.4) | 2.9 (4.3) | 0.345 |
|  | Other | 4.4 (4.5) | 4.1 (4.4) | 6.2 (4.9) | <.001 | 3.3 (4.5) | 2.8 (4.0) | 6.7 (6.0) | 0.021 |

**Note.** *n* unweighted; means (SD) weighted. NH = non-Hispanic. “Other” includes American Indian/Alaskan Native and multiracial individuals.
